# Identification and mapping of quantitative trait loci (QTL) conferring resistance to *Fusarium graminearum* from soybean PI 567301B

**DOI:** 10.1007/s00122-015-2473-5

**Published:** 2015-02-18

**Authors:** Bhupendra Acharya, Sungwoo Lee, M. A. Rouf Mian, Tae-Hwan Jun, Leah K. McHale, Andrew P. Michel, Anne E. Dorrance

**Affiliations:** 1 Department of Plant Pathology, The Ohio State University, 1680 Madison Avenue, Wooster, OH 44691 USA; 2 Department of Entomology, The Ohio State University, 1680 Madison Avenue, Wooster, OH 44691 USA; 3 Present Address: Department of Crop Science, North Carolina State University, 3127 Ligon Street, Raleigh, NC 27607 USA; 4 USDA-ARS and Department of Horticulture and Crop Science, The Ohio State University, 1680 Madison Avenue, Wooster, OH 44691 USA; 5 Present Address: USDA-ARS, Soybean Nitrogen Fixation Unit, Raleigh, NC 27606 USA; 6 Present Address: Department of Plant Bioscience, Pusan National University, Busan, 609-735 South Korea; 7 Department of Horticulture and Crop Science, The Ohio State University, 2021 Coffey Road, Columbus, OH 43210 USA

## Abstract

**Key message:**

**A major novel QTL was identified in a recombinant inbred line population derived from a cross of ‘Wyandot’** **×** **PI 567301B for** ***Fusarium graminearum,*** **a seed and seedling pathogen of soybean**.

**Abstract:**

*Fusarium graminearum* is now recognized as a primary pathogen of soybean, causing root, seed rot and seedling damping-off in North America. In a preliminary screen, ‘Wyandot’ and PI 567301B were identified with medium and high levels of partial resistance to *F. graminearum*, respectively. The objective of this study was to characterise resistance towards *F. graminearum* using 184 recombinant inbred lines (RILs) derived from a cross of ‘Wyandot’ × PI 567301B. The parents and the RILs of the mapping population were evaluated for resistance towards *F. graminearum* using the rolled towel assay in a randomized incomplete block design. A genetic map was constructed from 2545 SNP markers and 2 SSR markers by composite interval mapping. One major and one minor QTL were identified on chromosomes 8 and 6, respectively, which explained 38.5 and 8.1 % of the phenotypic variance. The major QTL on chromosome 8 was mapped to a 300 kb size genomic region of the Williams 82 sequence. Annotation of this region indicates that there are 39 genes including the *Rhg4* locus for soybean cyst nematode (SCN) resistance. Based on previous screens, PI 567301B is susceptible to SCN. Fine mapping of this locus will assist in cloning these candidate genes as well as identifying DNA markers flanking the QTL that can be used in marker-assisted breeding to develop cultivars with high levels of resistance *to F. graminearum*.

## Introduction

The incidence of seedling diseases in soybean [*Glycine max* (L.) Merr] caused by both oomycete and true fungi has increased substantially as the number of acres and the number of fields in continuous soybean has increased (Broders et al. 2007; Ellis et al. 2011). *Phytophthora sojae* and *Pythium* spp. have long been known in Ohio for association with seed and seedling diseases causing pre- and post-emergence damping-off in soybean and are responsible for severe production losses in the state (Schmitthenner 1985). In addition to these, another residue-borne pathogen, *Fusarium graminearum* [telemorph: *Gibberella zeae* (Schwein) Petch] has also been identified as a primary pathogen of soybean causing seed rot and seedling damping-off in North America (Broders et al. 2007; Díaz Arias et al. 2013a; Xue et al. 2007).

Symptoms of seed rot and seedling damping-off caused by *F.* *graminearum* appear first as water soaked lesions followed by light brown or pink discoloration around the inoculation point. At later stages seed, seedling, and root rots develop (Broders et al. 2007; Ellis et al. 2011; Xue et al. 2007). In South America, infections can also occur at pod filling and symptoms include spreading of discoloration vertically on the stem, interveinal chlorosis of leaves leading to plant wilting, pod blight and death (Martinelli et al. 2004; Pioli et al. 2004).

*F. graminearum* is a homothallic, ascomycete fungus and its sexual reproduction involves the development of perithecia that produce ascospores. This fungus produces macroconidia as asexual spores but lacks microconidia (Leslie and Summerell 2006). The macroconidia are slender and elongated, containing 3–5 cells separated by septa and play an important role in the dissemination of the pathogen (Harris 2005; Leslie and Summerell 2006). In addition to *F. graminearum*, many closely related species were also found to cause disease, hence, the fungi that contribute to these infections are now known as the *Fusarium graminearum* species complex (FGSC) (O’Donnell et al. 2004; Starkey et al. 2007; Taylor et al. 2000; Wang et al. 2011). In the United States, *F. graminearum* sensu stricto is the predominant species causing head blight (Gale et al. 2007). *Fusarium graminearum* has been more extensively studied as a pathogen of wheat and barley, which caused estimated yield losses of close to $3 billion during the scab epidemics in the US in the 1990s (Windels 2000). More importantly, *Fusarium* isolates with wide differences in aggressiveness have been detected, but no biological races have been reported to date in the *Fusarium* head blight––wheat system (review by Buerstmayr et al. 2009). This residue-borne pathogen also causes *Gibberella* ear and stalk rot of corn. In addition to causing yield loss, they produce mycotoxins, secondary metabolites, such as deoxynivalenol (DON), nivalenol (NIV) and zearalenone (ZEN). Type B trichothecenes (DON and NIV) are produced by every species in the *Fusarium graminearum* species complex (O’Donnell et al. 2008), are toxic to humans, animals, plants, insects and other microorganisms and are also important in fungal pathogenesis and antagonism (Mule et al. 2005).

Numerous quantitative trait loci (QTL) for resistance towards *F. graminearum* have been identified in both wheat and corn. Interestingly, different QTL have been identified for different types of diseases caused by the same pathogen, and for toxin production (Somers et al. 2003; Tamburic-Ilincic et al. 2009). For instance, QTL controlling seedling blight, head blight and DON accumulation, caused by *F. graminearum*, were different in wheat (Somers et al. 2003; Tamburic-Ilincic et al. 2009). In corn, *F. graminearum* can infect ears through the silk channel or through wounds made by birds and insects damaging the husk tissue and grain (Chungu et al. 1996; Reid and Hamilton 1996; Reid et al. 1992a, b). Previous studies identified quantitative resistance loci for silk or kernel resistance; the former prevents the fungus from growing down from silk to kernel and the latter prevents spread of the fungus from kernel to kernel when silk resistance is ineffective (Ali et al. 2005; Chungu et al. 1996; Reid et al. 1992a, b). Ali et al. (2005) detected 11 QTL for silk resistance and 18 QTL for kernel resistance in corn of which only two were the same for both silk and kernel resistance. Similarly, Martin et al. (2011) identified six major effects QTL that significantly enhanced ear rot resistance, including four QTL that significantly lowered DON accumulation and five QTL that contributed to lower zearalenone levels. Quantitative resistance has also been identified to *Gibberella* stalk rot in corn (Pè et al. 1993; Yang et al. 2010) including a single resistance gene, *Rfg1* (Yang et al. 2004).

*F.* *graminearum* was found to be highly aggressive in causing severe root rot in soybean compared to other *Fusarium* species (Broders et al. 2007; Díaz Arias et al. 2013a; Ellis et al. 2011; Zhang et al. 2010). In Ohio, *F. graminearum* was readily isolated from soybeans with symptoms of seedling blight collected in the field (Broders et al. 2007). *F. graminearum* was also the most frequently recovered species of *Fusarium* in fields in Iowa (Díaz Arias et al. 2013b) and in Eastern Ontario (Zhang et al. 2010). Since corn and wheat are often grown in rotation with soybean in the north central region of the USA, and because of the increased reports of this species contributing to seedling blights and root rots, it is important to examine potential risk of this pathogen and to establish disease management strategies.

A combined total of more than 250 soybean genotypes were screened with *F. graminearum* in three previous studies that readily identified soybean genotypes with resistance (Acharya 2014; Ellis et al. 2012; Zhang et al. 2010). However, genetic analysis of this resistance has thus far been limited to only one study (Ellis et al. 2012). They reported five QTL conferring resistance to *F. graminearum*, of which resistance alleles were contributed from ‘Conrad’ for four loci and from ‘Sloan’ for the other (Ellis et al. 2012). Among the 200 cultivars and plant introductions screened for resistance by Acharya (2014), plant introduction (PI) 567301B has high levels of resistance and ‘Wyandot’ has moderate level of resistance towards *F. graminearum*. A recombinant inbred line (RIL) population derived from a cross of Wyandot × PI 567301B had been developed and used to map QTL associated with resistances to soybean aphid (*Aphis glycines*) and powdery mildew (*Microsphaera diffusa*) (Jun et al. 2012a, b). A high density genetic map of this mapping population was also constructed recently (Lee et al. 2015). Therefore, the objective of this study was to identify QTL for resistance to *F. graminearum* using this same Wyandot × PI 567301B-derived RIL population.

## Materials and methods

### Plant materials

A subset of 184 *F*_7:10_ RILs derived from the cross of Wyandot × PI 567301B was used to map QTL associated with resistance towards *F. graminearum*. A high-density linkage map was completed on the full population of 357 *F*_7:10_ RILs of the same population (Lee et al. 2015). The cross was made in 2006 at Ohio Agricultural Research and Development Center (OARDC), Wooster, OH and advanced by single seed descent to develop RILs used in this study. Wyandot (OARDC–OSU) is a maturity group (MGII) soybean cultivar and PI 567301B is a MG IV accession originally collected from Gansu, China. DNA was extracted from young leaf tissue from each RIL as previously described (Lee et al. 2015).

### Inoculum production

Inoculations were carried out with *F. graminearum* isolate Fay 11, originally collected from soybean and previously shown to be highly aggressive when compared to several other isolates (Ellis et al. 2011). Macroconidia of this isolate were stored at −80 °C in 10 % glycerol in cryovials (Corning, New York) until used. Macroconidia were transferred to Petri plates with mungbean agar and grown for 10 days under fluorescent light with a 12 h light/dark cycle. Plates were then flooded with 5–10 ml sterile distilled water, macroconidia were dislodged with a glass rod and the resulting suspension was filtered through 4–6 layers of cheese cloth. The final macroconidia suspension was measured using a hemacytometer (Bright-Line Hemacytometer, Hausser Scientific, Horsham, PA) and sterile distilled water was added for a final concentration of 2.5 × 10^4^ macroconidia ml^−1^. Inoculation of seed with this concentration of macroconidia had the greatest disease severity in rolled towel assays (Ellis et al. 2011) and the same concentration was used to map QTL for resistance to *F. graminearum* in a different population (Ellis et al. 2012).

### Phenotypic evaluation

A rolled towel assay was used as described by Ellis et al. (2011) with slight modifications. Fifteen seeds from each RIL and checks were placed on a germination paper moistened with deionized water and inoculated with a 100 µl suspension of 2.5 × 10^4^ macroconidia ml^−1^. A second moistened towel was placed over the inoculated seeds, rolled up and placed on the top of a wire mesh in a 25 l bucket. The buckets were covered and stored in the dark at room temperature (~23–25 °C). Ten days after inoculation (dai), lesion length and total plant length were measured for each seedling of each genotype. A disease severity index (DSI) was calculated by dividing lesion length by total length of the seedlings and multiplying by 100. Disease severity was also assessed by a score using a 1–5 scale where, 1 = germination and healthy seedlings with no visible signs of colonization; 2 = germination and colonization of the root with 1–19 % of the root with lesions; 3 = germination and colonization of the root with 20–74 % of the root with lesions; 4 = germination and complete colonization of the root with 75 % or more of the seedling rot with lesions and 5 = no germination and complete colonization of the seed.

The 184 *F*_7:10_ RILs were divided into two groups of 92 RILs each and evaluated separately. Each group of 92 RILs was evaluated in an incomplete block which consisted of 40–48 RILs and, the two parents of the mapping population, ‘Conrad’, ‘Williams’ and ‘Sloan’ as checks in each individual assay. It took 2–3 assays to complete one full rep, and this was repeated two more times so that each RIL was evaluated three times.

The mean DSI and disease score of each RIL was calculated from 15 seedlings in each rolled towel in each replication of each experiment. Since the mean DSI and disease score were very similar, only DSI data was used for the following statistical analyses. DSI data from each replication were analysed to obtain the best linear unbiased predictor (BLUP) (Stroup 1989) using proc mixed procedure of SAS version 9.3 (SAS Institute Inc., Cary, NC, USA). The model applied in the analysis was *Y*_ijklm_ = *μ* + *G*_i_ + *R*(*G*)_ij_ + *S*(*RG*)_ijk_ + *C*_l_ + *L*(*C*)_lm_ + *ε*_ijklm_ where, *μ* is overall mean, *G*_i_ is the effect of *i*th group, *R*(*G*)_*ij*_ is the effect of *j*th replication in *i*th group, *S*(*RG*)_ijk_ is the effect of *k*th set in *j*th replication in *i*th group, *C*_l_ is the effect of *l*th class of entry (*l* = 1, 2, 3, 4, 5 and 6 for Conrad, Sloan, Williams, Wyandot, PI 567301B and RILs, respectively), *L*(*C*)_lm_ is the effect of *m*th genotype within class for recombinant inbred lines only (genotypic variance, $\sigma_{\text{g}}^{2}$) and *ε*_ijklm_ is the experimental error (*σ*_2_). Class of entry was treated as a fixed effect; all other terms were treated as random effects. The heritability was calculated as: $\sigma_{\text{g}}^{2} /(\sigma_{\text{g}}^{2} + \sigma^{2} /r)$, where *r* is the number of replications per RIL.

### Genotypic assay

Single nucleotide polymorphism (SNP) genotyping was performed using the Illumina Infinium BARCSoySNP6k BeadChip and genotypes were scored as described in Lee et al. (2015), where a high-density genetic map with 2545 SNPs based on 357 RILs of Wyandot × PI 567301B population was reported. For the present study, the genetic map was generated using JoinMap4 (Van Ooigen 2006) based on only the subset of 184 RILs used in the phenotypic assay. Two SSR markers (Sat_157 and Sat_162) on chromosome 8 were also added to increase marker density in the genomic region of the *F. graminearum* resistance QTL identified in this study. Polymerase chain reactions (PCR) for the SSR markers were performed as previously described (Ellis et al. 2012) using genomic DNA (50 ng) as a template in a volume of 12.5 µl and amplicons were visualized under ultraviolet light by electrophoresis through 4 % high resolution agarose gels (Amresco, Solon, OH) and prestained with GelRed (Biotium Inc., Hayward, CA in 1× RapidRun buffer (USB, Cleveland, OH). For QTL identification, composite interval mapping (CIM) was conducted using MapQTL^®^ 5 (Van Ooigen 2004). For CIM, the significance threshold was determined by a 1000-permutation test at *α* = 0.05 (Churchill and Doerge 1994).

## Results

### Phenotypic evaluation

**Fig. 1:**
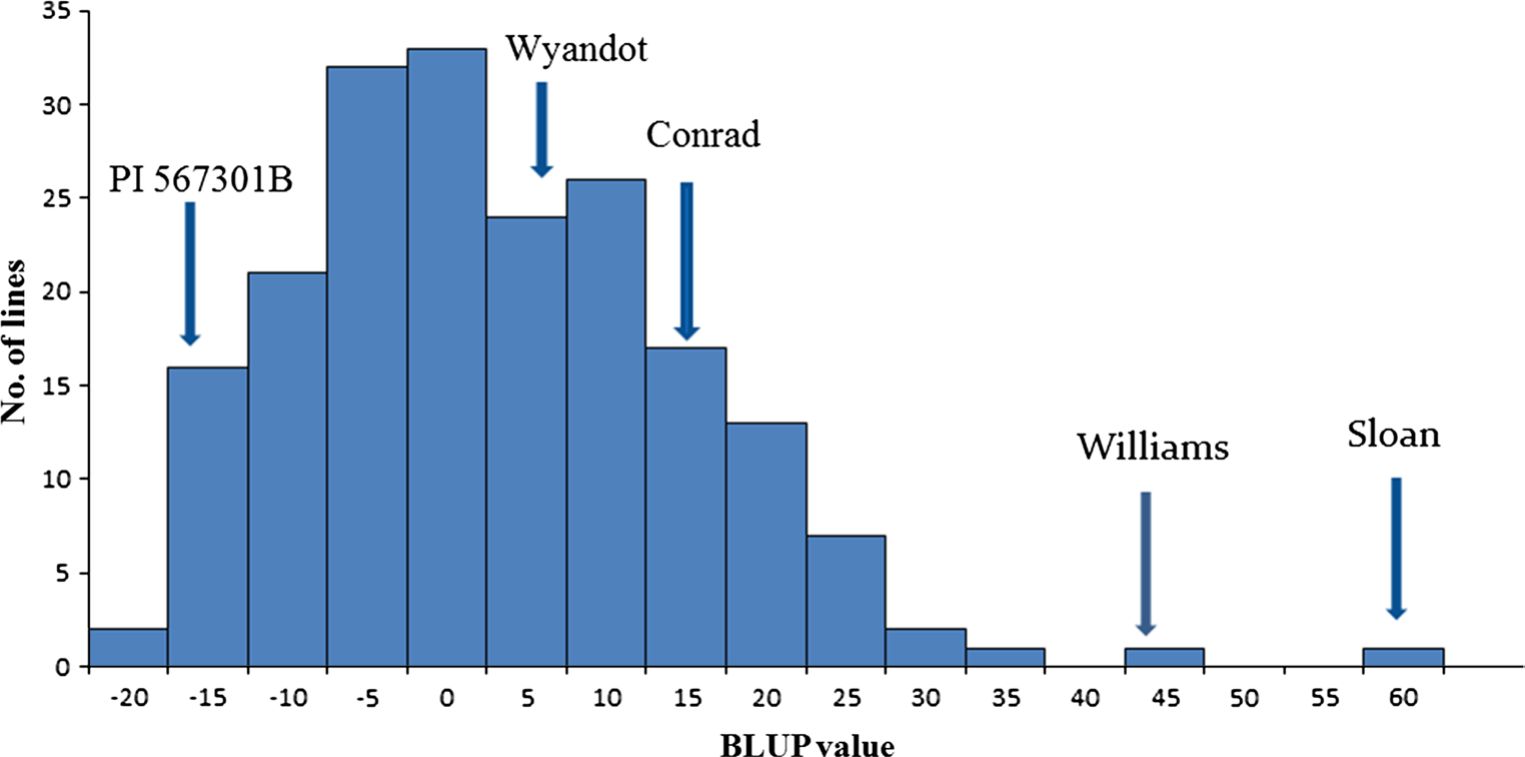
Frequency distribution of BLUP values for disease severity index of *F*_7:10_ recombinant inbred lines (RILs) population derived from the cross of ‘Wyandot’ × PI 567301B. Estimates of two parents and checks are indicated by *arrows*. A lower BLUP value means higher level of resistance to *F. graminearum*

The mean DSI was significantly different among the two parents and the checks indicating that PI 567301B had a high level of resistance and Wyandot had a moderate level of resistance towards *F. graminearum* (Table 1). The mean DSI and root rot scores of parents and checks were significantly different from one another (*P* < 0.0001; Fisher’s protected least significant difference test) (Table 1). The mean DSI of individual RILs ranged from 0.8 to 63.7 %, and the overall mean DSI of all the RILs was 32.6 %. Both Wyandot and PI 567301B had BLUP values less than Conrad, a moderately resistant cultivar, indicating that these parents had a higher level of resistance to *F. graminearum* than Conrad and that this was a moderately resistant by resistant cross (Fig. 1).

**Table 1:** Mean disease severity index (DSI) and mean disease score of the checks and RILs used in a rolled towel assay to evaluate resistance towards *Fusarium graminearum* in 184 RILs derived from a cross of ‘Wyandot’ × PI 567301B

Checks	DSI	Grouping^a^	Disease score^b^	Grouping^a^
Sloan	87.4	A	4.3	A
Williams	66.4	B	3.7	B
Conrad	43.1	C	3.0	C
Wyandot	35.7	D	2.8	D
PI 567301B	8.6	E	1.6	E

^a^Grouping is based on Fisher’s protected least significant difference test at *P* < 0.05

^b^Disease score is based on a disease rating, using 1–5 scale: 1 germination and healthy seedlings with no visible signs of colonization, 2 germination and colonization of the root and 1–19 % of the root with lesions, 3 germination and colonization of the seeds, and 20–74 % of the root with lesions, 4 germination and colonization of the seed and 75 % or more of the seedling rot with lesions, 5 no germination and complete colonization of the seed

The BLUP values calculated for DSI were normally distributed in this population (Fig. 1). Lower BLUP values indicate higher level of resistance to *F. graminearum.* Using the mixed model analysis, the BLUP values were estimated −17.3 and 9.6 for PI 567301B and Wyandot, respectively. The BLUP values of the checks Conrad, Williams, and Sloan were estimated to be 17.0, 40.4, and 59.9, respectively. Six RILs had BLUP values lower than PI 567301B and 38 RILs had BLUP values higher than Wyandot. The broad-sense heritability for the mean DSI was 0.79.

### Genetic linkage map

Genotyping with the BARCSoy6K BeadChip array and the two SSR markers included on Gm08 resulted in integrating a total of 2547 markers in 20 linkage groups, corresponding to the 20 chromosomes of soybean. Total length of this genetic linkage map was 2,342 cM and marker order was comparable with the genetic map of 357 RILs described in Lee et al. (2015). The average marker interval ranged from 0.9 to 1.6 cM by chromosome with the overall average marker distance of 1.2 cM. Based on physical positions of the SNPs, each linkage map covered 94.4–99.8 % of each chromosome.

### QTL identification

**Fig. 2:**
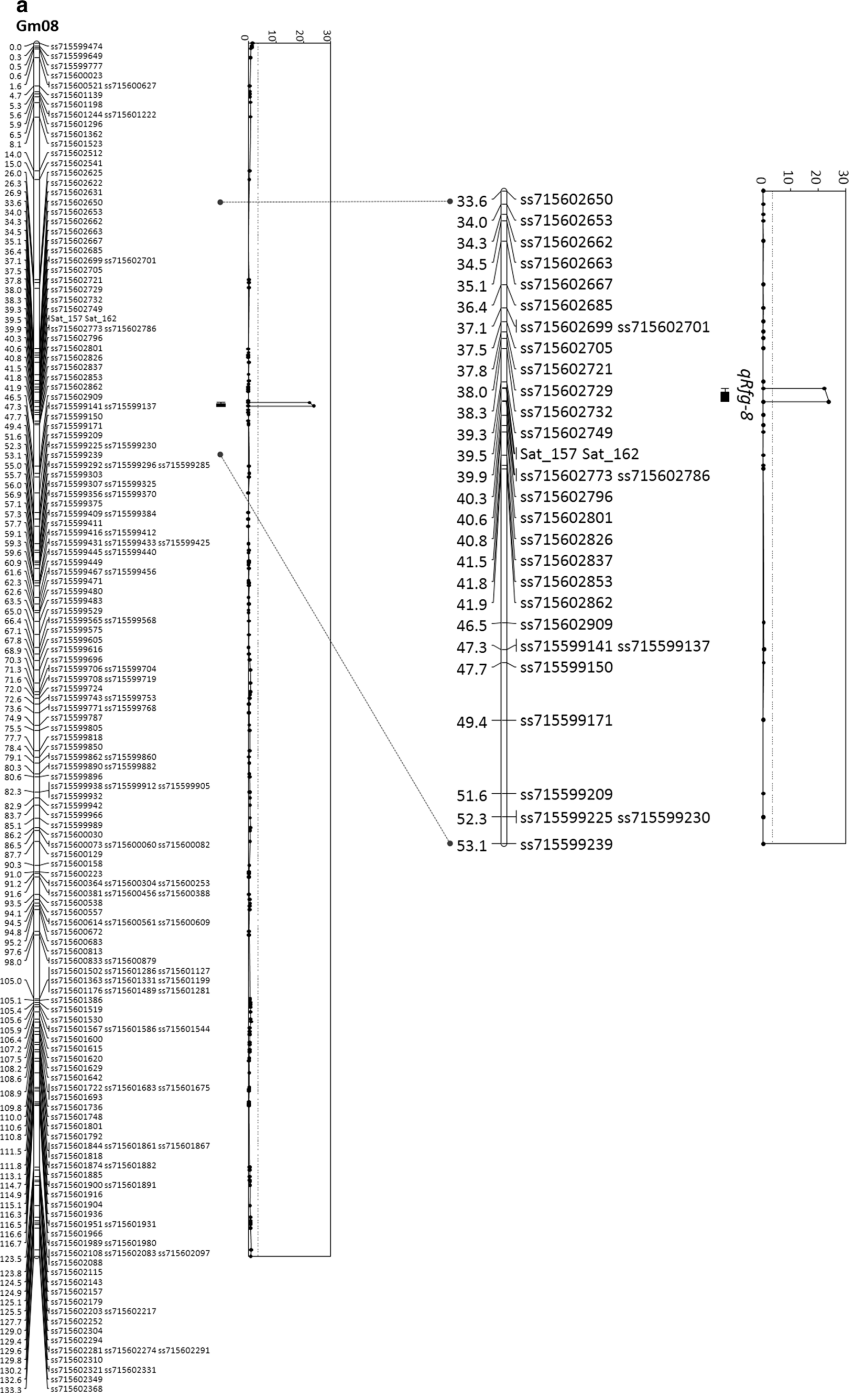

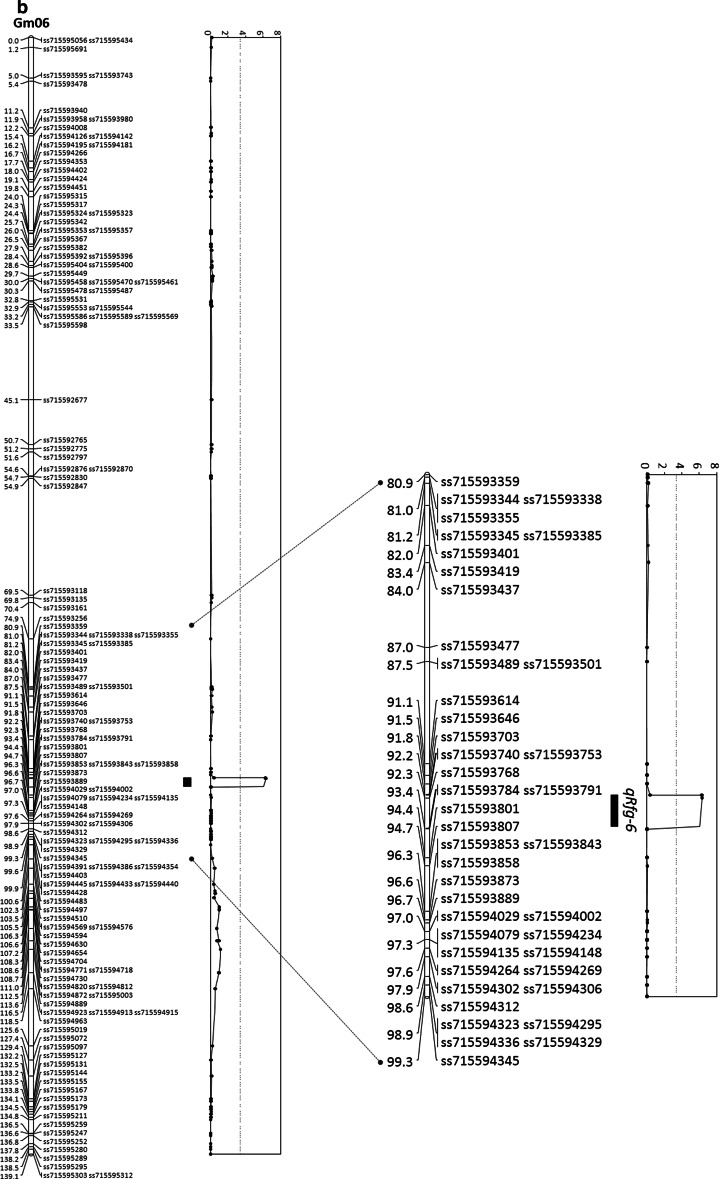
Graphical presentation of quantitative trait loci (QTL) for resistance to *Fusarium graminearum* identified on chromosome **a** 8 and **b** 6 in the ‘Wyandot’ × PI 567301B population by genome-wide LOD threshold 3.4 (a *hatched line* in LOD plots). The 1- and 2-LOD intervals are presented by a *black bar* and *solid lines* between the chromosome and the LOD plot for each QTL. The enlarged image was presented side by side to highlight approximate 20 cM region around LOD peak of each QTL

Two QTL conferring resistance to *F. graminearum* were identified in the Wyandot × PI 567301B population by CIM, one major QTL on chromosome 8 and one minor QTL on chromosome 6 (Table 2). Both QTL were highly significant with respective LOD scores of 23.9 and 6.3 compared to the genome-wide LOD threshold of 3.4 calculated by a 1000-permutation test. The major QTL on chromosome 8 was flanked by markers Sat_157 and ss715602786 and accounted for 38.5 % of the phenotypic variance (Table 2; Fig. 2a). A minor QTL, which explained 8.1 % of the phenotypic variance, was closely linked to marker ss715593768 (Glyma.Wm82.a2, Gm06 at 17672411 bp) on chromosome 6 (Table 2; Fig. 2b). The resistance alleles for both QTL were contributed by PI 567301B (Table 2).

**Table 2:** Quantitative trait loci conferring resistance to *Fusarium graminearum* identified via composite interval mapping in the ‘Wyandot’ × PI 567301B population

Chr.^a^	Peak marker (physical position)^b^	QTL interval (physical position)	LOD^c^	PVE (%)^d^	Additive effect^e^
6	ss715593768 (17,672,411)	ss715593740…ss715593784 (17,401,316…18,230,296)	6.3	8.1	3.16
8	ss715602773 (8,571,552)	Sat_157…ss715602786 (8,353,754…8,657,875)	23.9	38.5	6.88

^a^Chromosome

^b^Marker with the highest LOD score at each locus and physical position is based on the second version genome assembly (Glyma.Wm82.a2)

^c^Log of odd. Genome-wide LOD threshold of 3.4 was determined by a 1,000-permutation test at *P* < 0.05

^d^Percentage of phenotypic variance explained by a QTL

^e^A positive additive effect indicates that the resistance allele is contributed by PI 567301B

Based on the current annotation of the ‘Williams 82’ sequence, Glyma.Wm82.a2.v1, in Soybase (www.soybase.org, accessed June 2014), the flanking markers on chromosome 8 encompassed a region of 305 kb in which 39 genes were annotated, many of which have a high potential as candidate genes for resistance to *F. graminearum.* Among these genes are those with functions related to a serine hydroxymethyl transferase (SHMT), a leucine-rich repeat, rapid alkalinzation factor (RALF); heat shock transcription factor, hydroxymethylglutaryl-CoA tranferase involved in chalcone and stilbene synthesis, two subtilisin-like proteases; a gene coding for lectins, and amino acid synthesis with aspartokinase-homoserine dehydrogenase as well as those with unknown function.

The minor QTL identified on chromosome 6 was flanked by markers ss715593740 and 715593784 (829-kb interval) and has 47 genes (www.soybase.org, accessed on August, 2014). Among these genes are at least four transcription factors, one leucine-rich repeat, several genes involved with initiation of translation and DNA repair as well as four genes with unknown functions.

## Discussion

We characterized the resistance towards *F. graminearum* in PI 567301B and identified a major QTL using a population with over 2,500 molecular markers. The major QTL detected on chromosome 8 is a novel QTL associated with resistance to *F. graminearum* and, due to the marker density, was narrowed to a 305-kb region on chromosome 8 using a RIL population derived from a cross of Wyandot × PI 567301B. The QTL which contributed a major effect (38.5 % of phenotypic variance) for resistance, potentially overlaps the *Rhg4* locus (www.soybase.org, accessed on July, 2014) located on chromosome 8 in the Soybean Consensus map 4.0 (Ariagada et al. 2012; Ferreira et al. 2011; Guo et al. 2006; Mahalingam and Skorupska 1995; Meksem et al. 2001; Vuong et al. 2011; Webb et al. 1995; Wu et al. 2009). Significant association of two molecular markers with resistance to three different isolates of *P. sojae* was also identified in this region on chromosome 8 (Wang et al. 2012). Interestingly, one of the minor resistance QTL for *F. graminearum* from the cultivar Conrad mapped near 102 cM (BARC-051847-11270) on chromosome 8 (Ellis et al. 2012), which is approximately, 56 cM (28 Mb) distant from the QTL identified in the present study.

Large effect QTL are atypical for race non-specific pathogens, although *F. graminearum* is now believed to behave as a hemibiotroph during infection of wheat heads (Brown et al. 2010; Trail 2009). As such, several major QTL have been identified for resistance to *F. graminearum* in wheat. The QTL *Fhb1* for resistance to spread of infection within wheat spikes explains up to 41.6 % of the variation and has been verified in numerous populations (Bai et al. 1999; Anderson et al. 2001; Buerstmayr et al. 2003; Pumphrey et al. 2007). Interestingly, wheat genotypes carrying *Fhb1* are able to detoxify DON (Lemmens et al. 2005). The DON detoxification reaction is carried out by uridine diphosphate (UDP)-glycosyltransferase. Subsequently, a gene putatively encoding UDP-glycosyltransferase, which was upregulated following inoculation in *Fhb1* lines, was proposed as a candidate for *Fhb1* (Schweiger et al. 2013). A third major QTL in wheat, *Qfhs.ifa*-*5A,* acts primarily through a reduction in the initial rate of infection and explained up to 24 % of the variation (Buerstmayr et al. 2003). A lipid transferase protein which is highly upregulated in a QTL-specific manner following inoculation represents candidate gene for this QTL (Schweiger et al. 2013). One QTL *qRfg1* for resistance towards *F. graminearum* infection of corn stalks contributed to reducing disease severity and accounted for over 30 % of variation (Yang et al. 2010). This QTL was fine mapped and has been proposed to encode a transcription regulator (Ye et al. 2013). A transcriptome analysis of maize near isogenic lines with or without *qRfg1* indicated that secondary metabolic pathways for biosynthesis of phenylpropanoids, biosynthesis of plant hormones, and biosynthesis of terpenoids and steroids may play important roles in resistance towards *F. graminearum* in corn roots and stalks (Ye et al. 2013).

Based on the Williams 82 annotated sequence (Glyma.Wm82.a2.v1), the interval to which the large effect QTL on chromosome 8 is located in the present study includes a nine-member family of genes putatively encoding hydroxymethylglutaryl-CoA, which is a key enzyme in flavonoid biosynthesis pathway and may indicate that secondary metabolite biosynthesis is also important in the defense response to *F. graminearum* in soybean. In addition, there are three annotated genes putatively encoding rapid alkanization factor (RALF) which can initiate a signal transduction pathway (Pearce et al. 2010). RALF peptides are present throughout the plant kingdom and have been shown to cause a loss of root growth inhibition in tomato seedlings (Pearce et al. 2010). Two genes encoding subtilisin-like proteases are also located within the region, which have a predicted function in recognition of cell wall components during the plant development and in the plant defense responses (Nelsen et al. 2004). Another candidate for a defense response is a gene encoding a brassinosteroid-regulated protein, which regulates the gene expression and promotes elongation in soybean (Zurek and Clouse 1994). These will all make excellent candidate genes for future studies that focus on expression and functional analyses to explore the mechanism of this resistance response to *F. graminearum.*

This QTL also contains a gene putatively encoding a serine hydromethyl transferase (SHMT), which was reported to function in photorespiratory pathway influencing resistance to biotic and abiotic stresses in Arabidopsis (Moreno et al. 2005). A study by Liu et al. (2012) confirmed that SHMT is the *Rhg4* gene using mutation analysis, gene silencing, and transgenic complementation techniques and also cloned the gene from soybean cultivar ‘Forrest’. Interestingly, Forrest was found to be susceptible to the same isolate of *F. graminearum* used in this study with a disease severity index of 70.7 % and mean disease score of 3.9 (Acharya 2014). PI 567301B is reportedly moderately susceptible and susceptible to SCN races 3 and 4, respectively (Germplasm Resources Information Network, www.ars-grin.gov, accessed July 2014). This indicates that while resistance to *F. graminearum* is potentially linked to *Rhg4*, the SHMT allele contributing to SCN resistance is unlikely to be involved in the resistance response to *F. graminearum*.

The minor QTL on chromosome 6 is also first identified for resistance to *F. graminearum* in the present study but has been reported for resistance to multiple biotic stresses in several previous studies. This QTL mapped close to the resistance QTL for sudden death syndrome (SDS) which is caused by *Fusarium virguliforme* (Kazi et al. 2008), *Sclerotinia sclerotiorum* (Huynh et al. 2010), *P. sojae* (Li et al. 2010), Phomopsis seed decay (Sun et al. 2013), soybean aphid (Jun et al. 2013) and corn earworm (Terry et al. 2000). Among the 47 genes within the genomic region defined by the markers flanking this QTL are four transcriptional factors, which are also good candidate genes for functional analysis for resistance to *F. graminearum*.

*Fusarium proliferatum* was also recently reported to be highly pathogenic to soybean (Díaz Arias et al. 2011). Therefore, future investigation is needed into the potential resistance from PI 567301B to other pathogenic species of *Fusarium* such as *F. proliferatum*, as it has been observed in wheat (Toth et al. 2008) and corn (Mesterházy 1982). Resistance towards *F. virguliforme* in PI 567301B was evaluated previously in a greenhouse assay and this accession was reported to be susceptible (Hartman et al. 1997). The occurrence of *F. graminearum* isolates containing 3 ADON (more aggressive compared to 15 ADON) and NIV chemotypes were reported recently in North America (Foroud et al. 2012; Gale et al. 2011; Starkey et al. 2007). Hence, future investigation into interactions between the chemotype of *F. graminearum* and resistance responses in these and other soybean genotypes is warranted. Albeit, a limited survey of *F. graminearum* chemotypes collected primarily from Iowa found the 15-ADON genotype (Ellis and Munkvold 2014).

To date, *F. graminearum* has only been reported as a root, seed and seedling pathogen in North America, however, the observation of pod blight in Brazil (Martinelli et al. 2004) and pod and seed infection in Argentina (Barros et al. 2012; Garcia et al. 2012) signals the possibility that infections in these later growth stages may occur in the areas where wheat, corn and soybean are grown in rotation and in fields with high inoculum levels and favorable environmental conditions. However, more studies are needed to determine if the same QTL will confer resistance to infections that occur at later soybean growth stages.

PI 567301B contains resistance alleles conferring resistance to at least three different pathogens and pests of soybean including *F. graminearum*, soybean aphid (Jun et al. 2012a), and soybean powdery mildew (Jun et al. 2012b) but is susceptible to *P. sojae* (Lohnes et al. 1996), *F.* *virguliforme* (Hartman et al. 1997), *Phakopsora pachyrhizi* (Miles et al. 2006), *Phialophora gregata* (Bachman et al. 1997), soybean cyst nematode (Noel, GRIN database), and moderately susceptible to *Macrophomina phaseolina* (Mengistu et al. 2013). Marker-assisted breeding to prevent or limit the introgression of susceptible alleles into elite germplasm will greatly enhance the rapid development of high-yielding adapted cultivars which are resistant to targeted pests and pathogens.

### **Author contribution statement**

Mr. B. Acharaya conducted phenotypic assays and co-wrote the manuscript, Dr. S. Lee constructed the genetic map and conducted the QTL analysis and co-wrote the manuscript. Dr. MAR Mian, conceived and oversaw development of the population, mapping design, and contributed to the analysis and map construction; Dr. T. Jun, development of population and earlier map construction, and Dr. L. K. McHale contributed to the writing and provided key information regarding the importance of these genomic locations. Dr. A. P. Michel, provided funding for the development of the populations, contributed to the writing. Dr. A. E. Dorrance (and United Soybean Board and Ohio Soybean Council) provided funding for the phenotyping study and analysis, conceived of the screening for resistance, oversaw phenotyping experiments, extensive guidance to BA and SL for completion of the study, and co-wrote the manuscript.
